# Clarifying questions about “risk factors”: predictors versus explanation

**DOI:** 10.1186/s12982-018-0080-z

**Published:** 2018-08-08

**Authors:** C. Mary Schooling, Heidi E. Jones

**Affiliations:** 1 Graduate School of Public Health and Health Policy, City University of New York, 55 West 125th St, New York, NY 10027 USA; 2 School of Public Health, Li Ka Shing Faculty of Medicine, The University of Hong Kong, Pok Fu Lam, Hong Kong Special Administrative Region China

**Keywords:** Risk factor, Predictor, Cause, Statistical inference, Scientific inference, Confounding, Selection bias

## Abstract

**Background:**

In biomedical research much effort is thought to be wasted. Recommendations for improvement have largely focused on processes and procedures. Here, we additionally suggest less ambiguity concerning the questions addressed.

**Methods:**

We clarify the distinction between two conflated concepts, prediction and explanation, both encompassed by the term “risk factor”, and give methods and presentation appropriate for each.

**Results:**

Risk prediction studies use statistical techniques to generate contextually specific data-driven models requiring a representative sample that identify people at risk of health conditions efficiently (target populations for interventions). Risk prediction studies do not necessarily include causes (targets of intervention), but may include cheap and easy to measure surrogates or biomarkers of causes. Explanatory studies, ideally embedded within an informative model of reality, assess the role of causal factors which if targeted for interventions, are likely to improve outcomes. Predictive models allow identification of people or populations at elevated disease risk enabling targeting of proven interventions acting on causal factors. Explanatory models allow identification of causal factors to target across populations to prevent disease.

**Conclusion:**

Ensuring a clear match of question to methods and interpretation will reduce research waste due to misinterpretation.

## Introduction

Biomedical research has reached a crisis where much research effort is thought to be wasted [1]. Recommendations to improve the situation have largely focused on processes and procedures, such as addressing high impact questions, starting from what is already known, registration of protocols, and making data available [1]. Here we additionally suggest that ensuring the conceptual approach matches the question would avoid conflating different questions and mistaking the answer to one question for the answer to a different question. Much observational biomedical research concerns the role of “risk factors” in disease, which is conducted for two main reasons, (1) risk stratification or prediction and (2) assessing causality. These are two fundamentally different questions, concerning two different concepts, i.e., prediction versus explanation, which require different approaches and have substantively different interpretations. However, the use of the term “risk factor” as something which may predict and/or explain means that these two concepts may be conflated so that a study may not fulfill either objective, i.e., neither predicts nor explains. For example, major predictors of cardiovascular disease have long been assumed to be targets of intervention [2]. After extensive and expensive research investment over more than 35 years, including the development, testing and failure of an entire new class of drugs (CETP inhibitors) [3], high density lipoprotein cholesterol has recently been identified to be a non-causal risk factor (i.e. predictor) for cardiovascular disease [4, 5]. Equally, factors that do not predict risk are very rarely identified as causal factors (such as factors that are ubiquitous in a given community and thus are not caught as increasing risk) [6], which suggests interventions are being missed. Given, the importance of avoiding ‘low priority questions’ [1], here, we clarify the difference between these two concepts and their use in observational studies.

## Prediction models

Risk stratification, prediction models or ‘weather forecasting’ models identify people or groups at high or elevated risk of a particular health condition, ideally so that they can be offered proven interventions, or other mitigation can be implemented. A very successful example of a risk stratification model is the Framingham score which predicts 10-year risk of heart disease in healthy people [7], to inform prevention, such as use of lipid modulators. Other examples include prognostic models for identifying the best cancer treatment [8], or models for predicting disease trends, such as Google Flu Trends [9]. These predictive models typically rely on statistical projections of previous patterns, and to be feasible usually rely on easily captured information. For example, the Framingham score can be applied in daily clinical practice, even in resource poor settings, because it only requires assessment of age, sex, smoking, blood pressure, lipids and diabetes, which are relatively cheap and quick to measure. Google flu trends was based on internet search terms for particular symptoms [9].

Prediction models are usually developed based on statistical criteria to fit the distribution of the data well, using techniques such as stepwise selection, or more recently machine learning techniques. Prediction models often include several “risk factors” to obtain a model that fits the data well and can explain the greatest amount of variance in the outcome health condition. The contribution of each “risk factor” is presented so that the reader can see the independent contribution of each one to the overall prediction, as well as measures of model fit. Prediction models are usually validated in similar populations. As with all statistical models they cannot be expected to predict well in novel circumstances [9], and are best developed using a representative sample of the population in which they will be applied. Consistently poor measurement will impair precision, because it adds noise. Inconsistently poor measurement will impair predictive power, because it may change the relation between risk factor and outcome. Prediction models may not be generalizable to populations that differ from the one in which they were developed because in a new population the correlation between predictive and true causal factors may be different. For example, the Framingham model often has to be calibrated to predict absolute risk of heart disease correctly in new populations [10]. While Google Flu trends is no longer providing estimates; it became inaccurate, possibly because the model needed dynamic recalibration of the relation between search terms and influenza to stay on track [9]. Tried and tested prediction models are immensely valuable for identifying target populations, i.e. people or groups in need of prevention or treatment, but the “risk factors” that predict a health condition are not necessarily targets of intervention. For example flu symptoms do not cause influenza, and are not targets of intervention to prevent the occurrence of flu. Whether the “risk factors” that predict health conditions in risk stratification models are also targets of intervention has to be established from different studies designed to assess effects of interventions. As such, it is not appropriate to calculate a population attributable risk or proportion for “risk factors” from a risk prediction model, because removal of these “risk factors” might or might not affect population health. Similarly, the purpose of predictive models is to explain the greatest amount of the variance in the outcome, so only factors that contribute to explaining the variance need to be included. The concepts of confounding, mediation and effect measure modification are not applicable to predictive models. Interaction terms can be added to predictive models to improve model fit, but these interactions should not be interpreted as indicating different effects by subgroup.

## Explanatory models

Explanatory models can be thought of as simplified, abstract, propositional models for some particular aspect of how the world works, which provide a guide as to how to manipulate items of interest. As such, explanatory models are based on potentially causal factors i.e., factors whose manipulation changes the outcome [11]. Studies assessing causality are explanatory rather than predictive. Explanatory models are designed to assess whether a particular “risk factor” explains the occurrence, or course, of disease and as such is a valid target of intervention. “Risk factors” selected as potential causal factors might be based on “risk factors” from predictive models, might be theoretically based or might be hypothesized from other sources. For example, the Framingham score includes factors, such as smoking and blood pressure, which undoubtedly cause heart disease, but also other “risk factors”, such as age, sex and high-density lipoprotein, whose causal role in heart disease is less clear [12]. In contrast, factors that do not predict disease might be identified as possible causal factors based on physiology or well-established theories. For example, observationally telomere length does not appear to predict renal cell cancer, but people with genetically longer telomeres are at greater risk [13], suggesting a causal role in renal cell cancer, as in other cancers [14].

Studies assessing the role of causal factors need to avoid the major sources of bias in observational studies designed to assess causality, which can be most simply thought of as confounding and selection bias [15, 16]. In addition, measurement error is often thought of as an additional source of bias, although non-differential measurement error usually biases towards the null and differential measurement error can be thought of as a form of selection bias. Confounding occurs when extraneous common causes of the putative cause and health condition are omitted so that a spurious relation is observed. For example, smoking causes both yellow fingers and lung cancer, so any assessment of the causal effect of yellow fingers on lung cancer would need to take smoking into account. Confounding is difficult to avoid unless all the common causes of the putative cause and disease are known. One of the simplest options, when experimental studies (randomized controlled trials) are not possible, is to use methods, such as Mendelian randomization, that are less open to confounding [17]. No method is assumption free, and Mendelian randomization has stringent assumptions, nevertheless it has clarified some controversies over the causes of cardiovascular disease, such as the role of high density lipoprotein-cholesterol [18]. A sufficient set of confounders needs to be identified from external knowledge of causality, measured accurately in the study and included in the analytic model, so that any residual confounding does not cause incorrect causal inference. Given, the difficulty of assessing both known and unknown confounders, demonstrating that estimates for other associations subject to the same confounding are coherent with known causal effects gives greater credence to any new estimates from the same study [19, 20]. For example, observational studies of hormone replacement therapy (HRT) in women found apparent benefits for accidents as well as for cardiovascular disease [21], which suggests residual confounding for cardiovascular disease because HRT would not be expected physiologically to protect against accidents. The apparently protective findings were also not coherent with estrogen having no benefit for men in the Coronary Drug project trial [22] and possibly causing myocardial infarction in young women [23]. Nevertheless, confounding can, potentially, be addressed in an observational study by collecting sufficient relevant information about the study participants, so that all confounding is accounted for by adjustment, inverse probability of treatment weighting or standardization.

Confounding is a causal concept and not relevant for predictive models [24]. Confounding cannot reliably be assessed from observational data; meaning that testing confounders for inclusion in an analytic model based on statistical correlations or changes in estimates is not valid. Conversely, factors that are not confounders should not be included in the analytic model because they may prevent assessment of the full effect of the hypothesized cause in question. For example, an observational study designed to assess the effect of alcohol on stroke should not include blood pressure in the model as a confounder. Blood pressure may cause stroke but is more likely a consequence of alcohol use than a cause of alcohol use, making blood pressure likely a mediator not a confounder. As such, adjusting the model for blood pressure would not give the full effect of alcohol on stroke. Studies designed to assess the role of potential causal factors should only present the effect estimate for the hypothesized cause in question, because estimates for other factors in the model are unlikely to be correctly controlled for confounding (sometimes referred to as the “Table 2 fallacy”) [25]. However, it may be helpful to present models adjusting for different sets of confounders because of the difficulty of unambiguously identifying confounders. Further, presenting both the crude and adjusted estimates for the hypothesized cause may elucidate the extent to which the effect estimate is influenced by the hypothesized confounders.

Selection bias occurs when the sample is inadvertently constructed in such a way as to generate a spurious relation, most often inadvertent selection on common effects of hypothesized cause and outcome [24], hence sometimes described as “collider bias”. For example a study assessing the relation of smoking with lung cancer in very old people might find no relation because the sample is by definition only those who have survived their smoking habit i.e., is dependent on smoking and not getting lung cancer [26]. Selection bias is difficult to detect because it may require conceptualizing the relation of hypothesized causal exposure with disease in the sample absent from the study. For example, a study assessing the relation of obesity with death in people with diabetes [27] will not give a valid causal estimate unless it takes into account the relation of obesity with death in the people with diabetes who are absent from the study because of illness or previous death. Similarly, a spurious link between potential cause and disease may arise from measurement dependent on potential cause and disease. Recovering from selection bias is only possible in certain circumstances, for example when external data is available, but cannot be guaranteed [16].

Biomedical “risk factors” in explanatory models are potentially causal, i.e., manipulating the “risk factor” changes the outcome, and like all causal factors, in everyday experience, would be expected to be consistent within their particular area of application, and hence generalizable (or more precisely transportable [11, 24]) to other situations. However, this consistency may not always be apparent or relevant, because not all parts of an explanatory model may be applicable in all situations. For example, an explanatory model for lung cancer could include smoking and asbestos, among other factors, but attempting to reduce lung cancer by manipulating smoking would not be effective in a non-smoking population. As such, consideration needs to be given as to how to apply the explanatory model so as to act on relevant causal factors in any given population [24]. It is appropriate to calculate population attributable risks or proportions for explanatory factors, because these are causal factors whose manipulation could impact population health. However, attributable risks or proportions tells us what proportion of the outcome would not have occurred had the exposure been absent, but does not guarantee that will be the effect of removing the exposure.

## Summary

Predicting and explaining risk of health conditions are answering two fundamentally different questions with completely different approaches and implications. In this context, researchers need to identify the intent or purpose of their study, as identifying who is at risk (risk stratification) or what would be an effective intervention (explanation), so as to ensure research questions are addressed appropriately and effectively. Some “risk factors” can be both predictors and explanatory factors, at the same, which may lead to conflation of these terms in the research community. For example, blood pressure is both a predictor and a cause of cardiovascular disease. However, studies where blood pressure was considered as a risk predictor would have a different purpose, research question and approach from studies where blood pressure was considered as an explanatory factor. Prediction and explanation typically require different approaches in terms of conceptualization, modelling, analysis, validation, presentation, interpretation, generalizability and risk attribution as summarized in Table 1. Risk prediction studies are using statistical techniques to generate contextually specific data-driven models requiring a representative sample that identifies people at risk of disease efficiently, but do not necessarily identify targets of intervention. Explanatory studies, ideally embedded within an explanatory model of reality, test causal factors that might be targets of intervention. Predictive models allow public health practitioners to identify populations at elevated risk of disease to enable targeting of proven interventions on causal factors. Explanatory models allow public health professionals to identify causal factors to target across populations to prevent disease.

**Table 1:** Attributes of predictive versus causal models

Attribute	Type of model	
	Predictive	Causal
Purpose	Risk stratification, risk prediction or “weather forecasting”	To test whether a factor or set of factors are causal
Type of model	Data-driven	Explanatory
Type of analysis	Data-driven selection procedure	Test of specific causal model
Role of risk factors	Jointly fit the distribution of the data	Potential targets of intervention
Attributes of typical risk factors	Cheap and easy to measure	Part of a causal model
Role of confounding	Confounding is a causal concept [24] so it is not relevant	Confounders, typically common causes of “risk factor” and health condition [24] need to be identified from external knowledge and their effect on the estimate removed, via adjustment or other means
Type of sample	Representative of the population in which the model will be applied	Free from selection bias for the association(s) of interest
Role of measurement error	Consistently poor measurement will impair precision. Inconsistently poor measurement will impair predictive power	Non-differential misclassification of the exposure or the outcome usually biases towards the null, differential will bias the estimates, measurement error for confounders impacts ability to appropriately adjust, measurement error for predictors of missingness impacts ability to approrpriately adjust for selection bias caused by loss to follow-up
Validation technique	Replication in a similar sample	Use of control exposures and outcomes [19]
		Coherence with high-quality estimates [20]
Presentation	Show the association of each “risk factor” with the outcome health condition	Only show the association of the “risk factor(s)” tested for causality with the outcome [25]
	Measures of model fit	Effect estimates and 95% confidence interval
Interpretation	Identification of those at risk of a specific outcome, ideally for preventive action	Identification of a potential cause of the outcome, whose modification might change the risk of the outcome
	Predictors of risk	Effects on risk
	Risk predictors should not be used to calculate population attributable fractions or risks because attribution implies causality when predictive models are not necessarily based on causal factors	Causal factors can be used to calculate population attributable fractions or risks because explanatory models are based on causal factors
Generalizability/transportability	Model may need to be recalibrated for use in a new population	May need to consider distribution of causal factors in a new population to get an estimate of the effect the causal risk factors on the population

## Conclusion

Explicitly distinguishing between the different purposes of observational biomedical studies and explicitly matching the approach, interpretation and wording to the researcher’s intent will enable more focused and productive use of research resources. Avoiding the imprecise term “risk factor” and using a word, such as ‘predictor’, in risk stratification studies and ‘explanatory’ factor in causal studies might bring clarity of thought and thereby reduce unwarranted assumptions in biomedical research.

## References

[1] Moher D (2016). Increasing value and reducing waste in biomedical research: who’s listening?. Lancet.

[2] Anderson KM (1991). Cardiovascular disease risk profiles. Am Heart J.

[3] Nicholls SJ (2018). CETP-inhibition and HDL-cholesterol: a story of CV risk or CV benefit, or both. Clin Pharmacol Ther.

[4] Keene D (2014). Effect on cardiovascular risk of high density lipoprotein targeted drug treatments niacin, fibrates, and CETP inhibitors: meta-analysis of randomised controlled trials including 117 411 patients. BMJ.

[5] Voight BF (2012). Plasma HDL cholesterol and risk of myocardial infarction: a mendelian randomisation study. Lancet.

[6] Ioannidis JP, Tarone R, McLaughlin JK (2011). The false-positive to false-negative ratio in epidemiologic studies. Epidemiology.

[7] Wilson PW (1998). Prediction of coronary heart disease using risk factor categories. Circulation.

[8] Turnbull AK (2015). Accurate prediction and validation of response to endocrine therapy in breast cancer. J Clin Oncol.

[9] Lazer D (2014). Big data. The parable of Google Flu: traps in big data analysis. Science.

[10] Liu J (2004). Predictive value for the Chinese population of the Framingham CHD risk assessment tool compared with the Chinese Multi-Provincial Cohort Study. JAMA.

[11] Pearl J (2009). Causality: models, reasoning, and inference.

[12] Musunuru K, Kathiresan S (2016). Surprises from genetic analyses of lipid risk factors for atherosclerosis. Circ Res.

[13] Machiela MJ (2017). Genetic variants related to longer telomere length are associated with increased risk of renal cell carcinoma. Eur Urol..

[14] Haycock PC (2017). Association between telomere length and risk of cancer and non-neoplastic diseases: a Mendelian randomization study. JAMA Oncol.

[15] Hernan MA, Hernandez-Diaz S, Robins JM (2004). A structural approach to selection bias. Epidemiology.

[16] Bareinboim BT, Pearl J. Recovering from selection bias in causal and statistical inference. In: Proceedings of the twenty-eighth AAAI conference on artificial intelligence; 2014. R-425.

[17] Smith GD, Ebrahim S (2003). ‘Mendelian randomization’: can genetic epidemiology contribute to understanding environmental determinants of disease?. Int J Epidemiol.

[18] Holmes MV, Ala-Korpela M, Smith GD (2017). Mendelian randomization in cardiometabolic disease: challenges in evaluating causality. Nat Rev Cardiol.

[19] Lipsitch M, Tchetgen E, Cohen T (2010). Negative controls: a tool for detecting confounding and bias in observational studies. Epidemiology.

[20] Schooling CM (2016). Concordance with known causal effects is a potential validity measure for observational studies. J Clin Epidemiol.

[21] Petitti DP, Sidney S. Postmenopausal estrogen use and heart disease. NEJM. 1986;315(131–2). 10.1056/NEJM198607103150213 3724801

[22] The Coronary Drug Project (1973). Findings leading to discontinuation of the 2.5-mg day estrogen group. The coronary Drug Project Research Group. JAMA.

[23] Petitti D (2004). Commentary: hormone replacement therapy and coronary heart disease: four lessons. Int J Epidemiol.

[24] Bareinboim E, Pearl J (2016). Causal inference and the data-fusion problem. Proc Natl Acad Sci USA.

[25] Westreich D, Greenland S (2013). The table 2 fallacy: presenting and interpreting confounder and modifier coefficients. Am J Epidemiol.

[26] Hernan MA, Alonso A, Logroscino G (2008). Cigarette smoking and dementia: potential selection bias in the elderly. Epidemiology.

[27] Tobias DK (2014). Body-mass index and mortality among adults with incident type 2 diabetes. N Engl J Med.

